# A noncoding RNA gene on chromosome 10p15.3 may function upstream of hTERT

**DOI:** 10.1186/1471-2199-10-5

**Published:** 2009-02-02

**Authors:** Norimasa Miura, Reina Sato, Tomoe Tsukamoto, Mika Shimizu, Hiroko Kabashima, Miho Takeda, Shunsaku Takahashi, Tomomi Harada, James E West, Harry Drabkin, Jose E Mejia, Goshi Shiota, Yoshikazu Murawaki, Arvind Virmani, Adi F Gazdar, Mitsuo Oshimura, Junichi Hasegawa

**Affiliations:** 1 Division of Pharmacotherapeutics, Department of Pathophysiological and Therapeutic Science, Faculty of Medicine, Tottori University, Yonago, Tottori 683-8503, Japan; 2 Department of S/M Cardiovascular Pulmonary Research, School of Medicine, Colorado University at Denver and Health Sciences Center, Denver, Colorado 80262, USA; 3 Department of S/M Medical Oncology, School of Medicine, Colorado University at Denver and Health Sciences Center, Denver, Colorado 80262, USA; 4 Development of human artificial chromosomes for gene therapy, Weatherall Institute of Molecular Medicine, Medical Science Division, Oxford University, Oxford, OX3 9DU, UK; 5 Division of Molecular and Genetic Medicine, Department of Genetic Medicine and Regenerative Therapeutics, Institute of Regenerative Medicine and Biofunction, Graduate School of Medical Sciences, Tottori University, Yonago, Tottori 683-8503, Japan; 6 Division of Medicine and Clinical Science, Department of Multidisciplinary Internal Medicine, School of Medicine, Tottori University Faculty of Medicine, Yonago, Tottori 683-8504, Japan; 7 Hamon Center for Therapeutic Oncology Research and Department of Pathology, University of Texas Southwestern Medical Center, Dallas, Texas 75235-8593, USA; 8 Department of Molecular and Cell Genetics, School of Life of Sciences, Faculty of Medicine, Tottori University, Yonago, Tottori 683-8503, Japan

## Abstract

**Background:**

We attempted to clone candidate genes on 10p14–15 which may regulate hTERT expression, through exon trapping using 3 BAC clones covering the region. After obtaining 20 exons, we examined the function of RGM249 (RGM: RNA gene for miRNAs) we cloned from primary cultured human hepatocytes and hepatoma cell lines. We confirmed approximately 20 bp products digested by Dicer, and investigated the function of this cloned gene and its involvement in hTERT expression by transfecting the hepatoma cell lines with full-length dsRNA, gene-specific designed siRNA, and shRNA-generating plasmid.

**Results:**

RGM249 showed cancer-dominant intense expression similar to hTERT in cancer cell lines, whereas very weak expression was evident in human primary hepatocytes without telomerase activity. This gene was predicted to be a noncoding precursor RNA gene. Interestingly, RGM249 dsRNA, siRNA, and shRNA inhibited more than 80% of hTERT mRNA expression. In contrast, primary cultured cells overexpressing the gene showed no significant change in hTERT mRNA expression; the overexpression of the gene strongly suppressed hTERT mRNA in poorly differentiated cells.

**Conclusion:**

These findings indicate that RGM249 might be a microRNA precursor gene involved in the differentiation and function upstream of hTERT.

## Background

Telomerase, which adds repeated telomere sequences to chromosome ends, is a ribonucleoprotein enzyme that includes an endogeneous RNA (hTR) which acts as a template and a reverse transcriptase (hTERT), and are crucial molecules [1-4]. Telomerase activity has been detected in immortalized and tumor cells and its strong expression represents an important difference between normal and cancer cells [5]. Although it is thought that telomere maintenance is predominantly due to telomerase activity [6,7], which is mainly regulated by hTERT expression in the presence of hTR [8,9], some gaps exist in the steps between immortalization, carcinogenesis, and cellular senescence [10]. Therefore, these unknown steps must first be fully identified before translational applications are possible [11-13]. As several reports have indicated that human chromosome 10p carries a set of genes involved in regulating telomerase activity [14-16], we performed microcell-mediated chromosome transfer, radiated microcell fusion, and bacterial artificial chromosome (BAC) transfer assuming loss of heterozygosity (LOH), and examined telomerase expression in transfectants to determine whether hTR- and hTERT-regulating genes are located on 10p15 [17]. After we determined the telomerase regulatory regions on chromosome 10p15.1 using DNA markers, we confirmed that the chromosomal fragments containing the regions induce senescence in the transfectants [18,19]. We then identified that radiated chromosomal fragments containing D10S1728 (a genomic region on chromosome 10p15) induced senescence in transfectants, and performed the sequencing of 3 BAC clones containing D10S1728 [20-22] (data not shown), exon trapping using the BAC clones [23-25], Northern blotting, and RT-PCR before cloning several full-length genes. One of the genes we cloned was named RGM249 (RNA gene for miRNAs which was 249 bp in length), and showed stronger gene expression in cancerous lesions than in normal liver.

We used the RNA interference (RNAi) method, which is a sequence-specific post-transcriptional gene silencing mechanism triggered by double-stranded RNA (dsRNA), to cause degradation of mRNAs homologous in sequence to dsRNA and to effectively inhibit gene-specific expression [26]. Because dsRNA transfection is a common strategy for suppressing genes of interest, the small interfering RNA (siRNA) technique is an excellent gene-specific method for determining the function of the targeted gene by inhibiting its expression [27-30]. After focusing on RGM249 (RGM: RNA gene for miRNAs; 249 for 249 bp) among the genes cloned on 10p14–15 for further analyses, we examined the telomerase regulatory function of RGM249 using the overexpressing cassette and the gene-specific siRNA.

In this report, we present a summary of our cloning research and introduce functional analyses regarding the identified gene regulating hTERT mRNA.

## Results and discussion

### Screening of BAC library and transfection into HMc-Li7

We investigated the 3 BAC clones using a sequence-tagged-site marker (D10S1728) by PCR, based on previous reports [18,19]. From these studies, we determined that the 3 BAC clones (M-6, H-11, and J-21) contained D10S1728.

### Whole sequencing of H-11 and exon trapping

**Figure 1:**
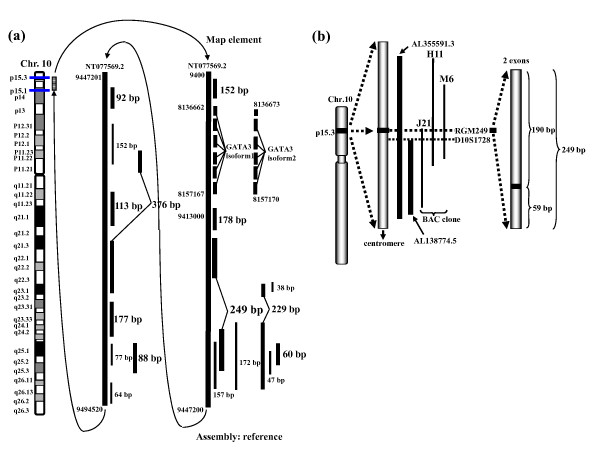
**Schematic diagram showing the chromosomal location (10p15.3) of exons including RGM249**. (a) Mapping of exons trapped between 10p15.1 and 10p15.3. Reference was used as an assembly database. This region does not contain any known genes except GATA3. Solid arrows indicate both ends of the sequences which BAC clones cover and show that 9447200 links to 9447201 in NT077569.2. (b) Schematic diagram showing the chromosomal location (10p15.3) of RGM249, which consists of 2 exons, genomic fragments (AL355591.3 and AL138774.5), and a genomic marker (D10S1728) containing the gene and BAC clones (H-11, M-6, and J-21) used for the mapping. The full length of the gene is 249 bp. Solid and dashed lines represent the exons trapped and their neighboring gene (GATA3), respectively.

Approximately 90%~95% of the BAC clone H-11 sequence and supercontig were determined (studies were performed by the University of Colorado Cancer Center Sequencing Core). D10S1728 was regarded as the sequence on 10p15.3. We reconfirmed that the sequence and supercontig were derived from the original BAC clones by Southern blotting and PCR, and 20 exons were thereafter identified (fig 1-(a)). We performed a computer analysis for each contig, and the endosequences of the 3 BAC clones and the exon sequences we trapped showed that they closely overlapped each other (fig 1-(b)).

### Northern blot analysis and one-step real-time RT-PCR

**Figure 2:**
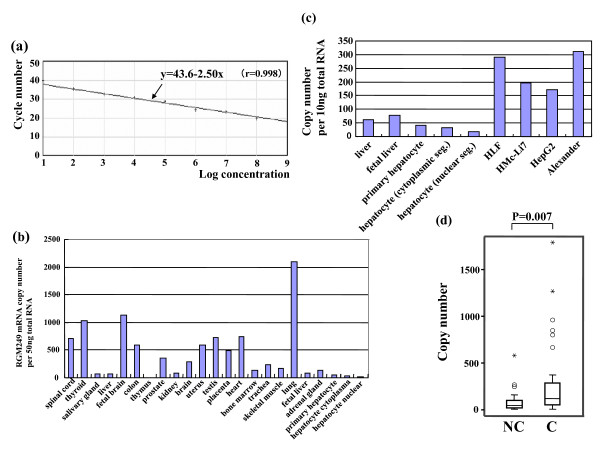
**A quantitative estimation in normal cells, cancer cells, and tissues**. To quantify RGM249 mRNA expression in hepatocytes, hepatoma cells, and liver tissues, the copy number in 10 ng of total RNA was evaluated. (a) One-step real-time PCR was performed based on the linear correlation (P > 0.990) of the RNA controls. (b) RGM249 mRNA expression was examined in human representative organs using one-step real-time RT-PCR. The vertical line shows the copy number per 50 ng/μl total RNA (c) RGM249 mRNA expression in liver-related cells (from the left: adult liver tissues, fetal liver tissues, primary cultured hepatocytes [T], cytoplasm segment of primary hepatocytes [C], nuclear segment of primary hepatocytes [N], HLF cells, HMc-Li7 cells, HepG2 cells, and Alexander cells). (d) RGM249 mRNA expression in surgically resected liver tissues containing 33 pairs of hepatomas and adjacent noncancerous tissue. C: cancerous lesion, NC: adjacent noncancerous lesion. Cancerous lesions showed a significant upregulation of RGM249 mRNA, compared with noncancerous lesions, as determined by the *t*-test (P = 0.007) and paired *t*-test (P = 0.040). Box represents 95% confidence intervals.

Using a probe against each exon sequence (47–376 bp), Northern blotting was performed, which demonstrated that several exons showed a very faint signal (data not shown). In particular, a very faint single hybridization signal in human Multiple Tissue Northern (MTN) blot (Clontech) migrating at about 250 bp was determined for RGM249. One-step real-time RT-PCR showed a very low expression rate, that is, less than 20,000 copies per 50 ng of total RNA extracted from human organs (fig 2-(b)). In normal liver, approximately 60 copies per 50 ng of total RNA of transcripts were expressed. In contrast, the study on human primary hepatocyte cultures, including nuclear and cytoplasmic compartments in 4 hepatoma cell lines (HepG2, Huh7, HMc-Li7 and Alexander), indicated that hepatoma cells expressed RGM249 at much higher levels than normal human hepatocytes (fig 2-(c)). In 39 hepatomas, the expression rate was higher (mean ± SEM: 609.2 ± 261.5 copy number per 100 ng/μl) than in the 35 adjacent noncancerous lesions (138.9 ± 46.5) (P = 0.007 by *t*-test and P = 0.040 by paired *t*-test) (fig 2-(d)).

### 5' & 3' RACE and cDNA library screening

**Figure 3:**
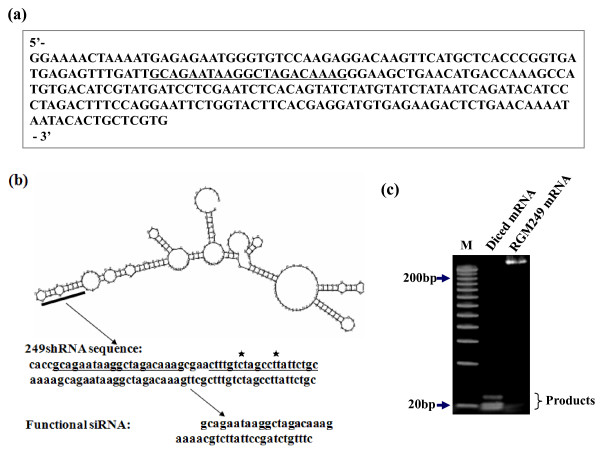
**Outlines of cloned RGM249, a possible miRNA precursor or telomerase regulated gene**. (a) RGM249 sequence is shown, and the sequences used for the siRNA and shRNA designs are underlined. (b) Predictive secondary structure of RGM249 was drawn by Vienna RNA Secondary prediction and comparison . Bold line corresponds to the sequence used as the most functional siRNA or shRNA. The underlined parts show the sense and antisense sequences generating shRNA. Star (⋆) shows deletion of T or C in mt-1RGM249 or mt-2RGM249, respectively. (c) From the digestion by RNase III (Dicer), RGM249 mRNA generates 3 products ranging from 17 to 23 bp, suggesting that this gene may function as a noncoding precursor RNA gene which produces miRNAs.

5' and 3' RACE was performed using liver c DNA libraries (Clontech). Screening of the cDNA libraries yielded a clone with a full sequence length of 249 bp. A comparison of the cDNA sequence with the genomic sequence from H-11 revealed that the gene of interest was extended across 9 kb. The cDNA named RGM249 consisted of 2 exons and the RNA was not poly-adenylated (fig 1-(b)). The sequence with about 40% of the GC ratio is shown in fig 3-(a), indicating that this gene might be a noncoding RNA (ncRNA) gene, as shown in fig 3-(b). PCR-Single Strand Conformation Polymorphism (PCR-SSCP) and sequencing showed that the gene had no mutations other than a one-base addition at the last site of exon 1 in 10 normal liver tissue samples (10%; 1/10) and no mutations in 4 tumor cell lines (0%; 0/4). Although the cDNA showed no homology with either additional human loci except for the endogeneous locus, or mouse genome, the miRNA predicted to be digested from RGM249 by Dicer matched at least 3 miRNAs in the miRNA database (miRBase; mature miRNAs or stem-loop sequences) . Although their functions remain unknown because we have not fully clarified whether RGM249 is actually processed by Dicer *in vivo*, given that it can be processed by Dicer *in vitro*. On the other hand, a motif search predicted no motifs in the RGM249 gene. Because this gene generated no protein, the secondary structure of RGM249 was drawn based on the structure provided at  (fig 3-(b)). Moreover, because the full-length RNA was digested by Dicer enzymes, 20–23 bp sharp bands could be observed, indicating that nucleotides generated from RGM249 RNA may be miRNAs (fig 3-(c)).

### GeneBank accession number

Sequence data have been deposited with the EMBL/GenBank Data Libraries under accession no. EF433558.

### The effects of downregulating RGM249

**Figure 4:**
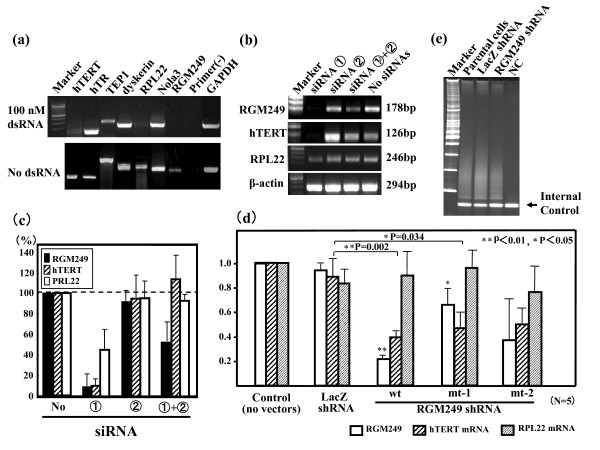
**Expression of telomerase-related genes in RNAi methods**. (a) Inhibitory effect of full-length RGM249 dsRNA (100 nM dsRNA) on the transcriptional expression of telomerase-related genes in a hepatocellular carcinoma cell line (HMc-Li7) as determined by RT-PCR. RGM249, hTERT, and RPL22 were significantly suppressed. The lower blotting demonstrates gene expression using a transfection reagent without dsRNA. (b) RT-PCR showing the inhibitory effects of RGM249-specific siRNA on RGM249 mRNA, hTERT mRNA, and RPL22 mRNA in HLF cells, which express RGM249 mRNA in 4 hepatoma cell lines in the strongest manner. siRNA ① and siRNA ② correspond to those in Table 2. (c) Quantitative evaluation of the suppressive effect of siRNA in which the measurement, in the case without siRNA, was regarded as 100% for standardization (N = 5), suggesting that siRNA ① has a functional sequence. RGM249 siRNA transcriptionally suppressed more than 80% of hTERT expression and approximately 50% of RPL22 expression.  RGM249 mRNA, ▯ hTERT mRNA,  RPL22 mRNA. d) Quantitative evaluation of the inhibitory effect of RGM249 shRNA on RGM249 mRNA, hTERT mRNA, and RPL22 mRNA. From left, transfection using only transfection reagents, LacZ shRNA as the gene control, wtRGM249 shRNA, and mt-1RGM249 or mt-2RGM249 shRNA with a different mutation (a nucleotide T or C) in the functional sequence. A total of 5 transfections were performed and data were statistically analyzed using the Mann-Whitney test. Compared with LacZ shRNA, RGM249 and mt-1RGM249 shRNA significantly suppressed hTERT mRNA (P = 0.002 and P = 0.034, respectively). mt-1RGM249 and mt-2RGM249 correspond to those in Table 2. ▯ RGM249 mRNA,  hTERT mRNA,  RPL22 mRNA, *: P < 0.05; **: P < 0.01. Quantitative evaluation of the suppressive effect of shRNA in which the measurement, in the case without siRNA, was regarded as 100% for standardization (N = 5). (e) Telomerase activity was quantitatively compared using image analyzer between transfectants with LacZ shRNA (in proliferative state) and RGM249 shRNA (in senesced state) and RGM249 shRNA, and was compared with the parental cells (112 and 54, respectively, which had an intensity of 100). Although the results depended on the timing of cell harvests after transfection, telomerase activity in transfectants with RGM249 shRNA was reduced to approximately half compared with that in parental cells, and we observed a periodicity of 6 bp due to reduced telomerase in the shRNA lane. NC: telomerase negative control.

In many hTERT- or hTR-regulating molecules reported previously, only two factors, hTERT and ribosomal protein L22 (RPL22), were transcriptionally suppressed in the hepatoma-derived cell lines (HLF) transfected with full-length RGM249 dsRNA (fig 4-(a))[31]. To reconfirm the findings in dsRNA method, the siRNA method was performed. Results showed that their expressions were suppressed in HLF cells transfected with Stealth siRNA ① (fig 4-(b),(c)).

RGM249 siRNA suppressed more than 90% of hTERT mRNA, approximately 50% of RPL22 mRNA, and RGM249 transcripts (fig 4-(c)) unlike hTR expression. However, RPL22 expression in cells transfected with Stealth siRNA ① was weaker (50%) than in those transfected with full-length dsRNA (fig 4-(c)). siRNA ① + ② had less efficient knockdown due to the interaction between both siRNAs. These findings are consistent with our results obtained from dsRNA induction, an experiment performed under conditions of almost negligible cytokine storm. A similar result was obtained about 48 hours after transfection.

Subsequently, we examined the inhibitory effect of shRNA transfection. Compared with LacZ shRNA, we found that RGM249 shRNA and mt-1RGM249 shRNA significantly downregulated hTERT mRNA (P = 0.002 and P = 0.034, respectively, using the Mann-Whitney test; fig 4-(d)). Otherwise, no significant differences were observed, except for the effect on RGM249 mRNA. Similar to transfectants that received RGM249 siRNA, telomerase activity in transfectants with shRNA was suppressed in the same manner as mRNA expression, compared with that in transfectants with LacZ shRNA (fig 4-(e)). Telomere length was not measured due to the small number of cells available and the relatively short observation period. No immunoreactive effects on tumor cell proliferation resulting from cytokine storm were recognized in the GE profile by microarray analysis, which resulted in IFN-β being downregulated twofold.

### The effects of overexpressing RGM249 mRNA

**Figure 5:**
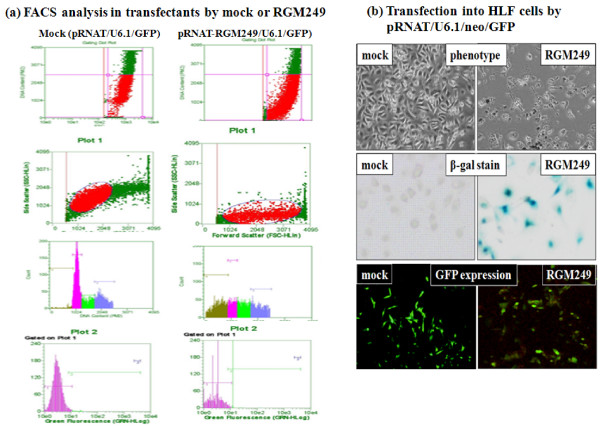
**FACS analysis and phenotypes in transfectants by RNAi methods**. (a) FACS analysis showing induction of more than half of the transfected populations into the preG1 phase of the cell cycle. As shown in the upper two parts, the increase in apoptotic cells in transfectants by RGM249 is shown as red circled areas. DNA content analysis revealed in the lower parts, that early cells showed typical cell proliferation profile, cells later in production showed reduced cell proliferation, and later cells also showed early signs of cell death (sub-G1), in both transfectants. In the bottom parts, the histogram with gating and the threshold were set properly for correctly stained cells in a clean machine. The large pink peak represents the green light signal from cells. The line segment flanking the peak is the gating band for cells. (b) Changes in phenotypes of HLF cells receiving LacZ shRNA or RGM249 shRNA are shown. The upper, middle, and bottom part shows the phenotype of transfectants, the result of β-Gal staining as an indicator of senescence, and EGFP expression as evidence of successful transfection, respectively. At 2–3 weeks after transfection, the transfectants were induced to undergo apoptosis (upper and bottom parts) through senescence (middle part) in the presence of both a selectable marker (neo) and RGM249 (data not shown).

In the transfection of HLF or A172 cells showing RGM249 overexpression, as distinct from the transfection of other cell lines such as senescing or well-differentiated cells, hTERT mRNA was significantly downregulated. In particular, the growth of A172 transfectants was strongly suppressed and they were induced to undergo senescence. On the other hand, hTERT mRNA was upregulated in KMST cells, which was established by exposure to chemical oncogens (Additional file 1). However, the expression of hTR, β-actin, and RPL22 mRNA showed no significant differences between mock transfectants and RGM249 transfectants in KMST cells, similar to the transcriptional level of c-Myc, undifferentiated markers (Rex-1 and GATA4), and an initiation factor (eIF6). Additionally, the longevity and proliferation rate of transfectants were almost the same as those of the parental cells or mock transfectants. Interestingly, we examined the effect of RGM249 shRNA wrapped by cationized carriers on tumorigenesis in nude mice using local or venous injection, which resulted in more than 60% of the suppressive effect *in vivo *(manuscript in preparation). FACS analysis showed that RGM249 mRNA expression introduced more than half of RGM249 pRNAT/U6.1/neo/GFP-transfected populations into the preG1 phase (fig 5-(a)), senescence, and apoptosis (fig 5-(b)), compared with pRNAT/U6.1/neo/GFP-transfectants. These phenotypic changes were similar to the effects of RGM249 shRNA, compared with those of LacZ shRNA

LOH in human hepatocellular carcinoma (HCC) is rare, except at 10q26.11-qter, which is observed in 25% of HCC cases (32). However, relatively frequent alterations and deletions of chromosome 10p have been identified in human tumors, including glioblastoma multiforme (GBM) and malignant melanoma [33,34]. In GBM, 3 common distinct regions of chromosome 10 deletion have been frequently identified; one of them is the distal half of the short arm [15,35], indicating that several distinct tumor suppressor genes may be present on chromosome 10 and may contribute to the progression of malignant tumors. Structural alterations have only been observed in malignant melanoma (10p13-pter), gastric cancer (10p15), and GBM (10p14-pter). Unexpectedly, RGM249 with low GC ratio on 10p15.3 is potently miRNAs-generating gene, as well as cancer-related gene.

**Figure 6:**
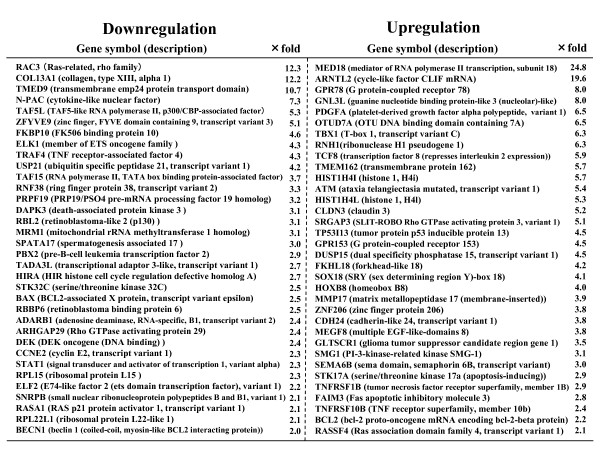
**A representative gene expression in microarray analysis**. Representatives of regulated genes using microarray analysis performed between transfectants with LacZ shRNA and RGM249 shRNA are shown as a gene symbol (description) and ×fold. Following transfection of the RNA generating-vector and presumably the generation of RNA molecules, many transcriptional factors, the molecules involved in RNA biogenesis, and tumor-related genes altered the expression level.

It is well known that a miRNA, generated from a miRNA precursor gene, can act as a new regulator which induces cancer cell lines to adopt a suppressive phenotype, except for the tumor suppressor gene. Because the phenotype we observed in a series of transfections with a chromosome (fragment) or BAC was suppressed, this miRNA might be a Suppressor-miR or it might have demonstrated a miRNA-directed inhibition. Actually, RGM249 repressed hTERT mRNA in A172 cells derived from GBM, moderately differentiated HCC (HMc-Li7), and poorly differentiated HLF. Beyond 60S RPL22, which we referred to in this study, several ribosomal protein mRNAs changed their expression level after the introduction of siRNA or shRNA into cell lines or tumors inoculated in nude mice (data not shown). In a miRNA-mediated mRNA repression study, RPL22, of which Greider et al. previously referred to as one of the binding protein to hTR that is essentially required for telomere maintenance and telomerase activity in normal tissues and disease, showed no significant alteration in expression level, whereas it showed an obvious change in siRNA-directed RNAi with reproducibility (2.08-fold downregulation in microarray analysis)[31]. We do not have any definite evidence that RPL22 functions in association with hTERT expression during carcinogenesis or in hypoxic tissues. However, while endogenous hTR expression is regulated at the transcriptional level, hTERT is regulated by alternative splicing under hypoxic conditions [36]. It may be that RPL22 influences hTERT expression through hTR expression. RGM249 dsRNA repressed RPL22. However, RGM249 siRNA and shRNA did not significantly repress RPL22 because of the high homology shared by 2 parts of the RGM249 sequence which are different from the functional sequence, which was used in siRNA or shRNA and corresponds to one part of the RPL22 sequence. Eukaryotic initiation factor 6 (eIF6) has long been known to bind to free 60S ribosomal subunits, preventing the assembly of translationally competent 80S ribosome particles [37]. Similar to eIF4E bound to Argonaute2 (Ago2), which regulates mRNA expression by cleaving its mRNA targets and competing with eIF6 for the mRNA cap [38], eIF6 may mediate siRNA-directed RNA interference rather than miRNA-directed translational repression. This study indicates that the sequence focused for siRNA or shRNA has a key function in this gene, and that RGM249 may functionally have a close link to gene-mediated processes at the transcriptional level, such as RNA biogenesis or gene regulation, and a link to ribonucleoproteins such as telomerase or RPL22. As a result, RNA molecules may induce suppressive or apoptotic events in cancer cells through an RGM249-related signaling pathway involved in a DNA-, RNA-, or protein-binding function (some of the significantly changed genes are shown in fig 6; there were 306 upregulated genes and 144 downregulated genes in the *in vitro *trial)

miRNA is physiologically generated by both Drosha and Dicer. We can not digest RGM249 mRNA by Drosha because Drosha protein is now unavailable commercially. RGM249 RNA processed by DICER, results in at least 3 small fragments of a product (17–23 bp) related to RNA species (fig 3-(c)). The sequencing of diced RGM249 mRNA proved that the sequence we used for siRNA or shRNA was included in fig 3-(b). Because the silencing of RGM249 induced the downregulation of hTERT expression, this gene may be involved in the oncogenic mechanism. In humans, ~800 miRNAs are predicted to exist [39]; each of these is thought to have hundreds of targets, suggesting that miRNAs play a critical regulatory role in cellular protein expression [40]. In various human cancers as well as in developmental timing and patterning, differentiation, organogenesis, and stress response, alterations of miRNA profiles have been observed; these miRNAs may act as oncogenes, leading to the specific dysregulation of their target gene transcripts [41,42]. Although alterations in miRNA expression have been described in cancer and may contribute to other disease states, it remains unclear what drives these alterations. Our findings might pave the way for defining unknown crucial roles that miRNAs located around this locus play in oncogenic regulation through hTERT regulation and, in particular, the role RGM249 may play during this process.

Although the way in which RGM249 regulates hTERT remains unidentified, the physiological expression of RGM249 synchronizes the upregulation of hTERT mRNA, and its excess expression using the RNA-expression vector downregulated hTERT in specific cancer cells. Moreover, its siRNA or shRNA suppressed hTERT. Taken together, RGM249 may function negatively or positively, reversibly or indirectly on unknown mediators upstream of hTERT. Using microarray analysis between transfectants with RGM249 siRNA and those with LacZ siRNA (fig 6), the comparative expression analysis demonstrated that ATM and TP53LP were upregulated, although p53 status (the presence of mutation) in each cell line did not correlate with hTERT mRNA expression, and Ras and several transcriptional factors were downregulated. As described in fig 6, RGM249 may have direct effects on several transcriptional factors. Rapid stress induced by siRNAs may induce upregulation of genes participating in both repairing action and apoptotic or growth inhibition induction. Comparative proteome analysis would be useful to explain the detailed mechanisms underlying these processes. The overexpression of RGM249 in normal cells did not alter hTERT expression level, suggesting that RNA-related molecules may be functionally different in normal cells and de-differentiated cells, and that a key molecule for uncovering the oncogenesis mechanism or a potential factor which could be applied as a therapeutic target may exist in the genome. The findings that both the overexpression and dsRNA knockdown of RGM249 suppressed the expression of hTERT are problematic. In addition, this gene may be indirectly associated with hTERT because its overexpression may not always reveal a normal function and RGM249 microRNAs are not predicted to bind to hTERT even with the use of available bioinformatics. Moreover, this gene is not mutated or homozygously deleted even in HCC cells and tissues. Based on the Celera assembly, it seems to have one base (T) addition at position 188 of the first exon, however, single nucleotide polymorphisms are not specific to cancer. Additional experiments such as promoter analysis are needed to elucidate the epigenetic network in which this gene works.

**Figure 7:**
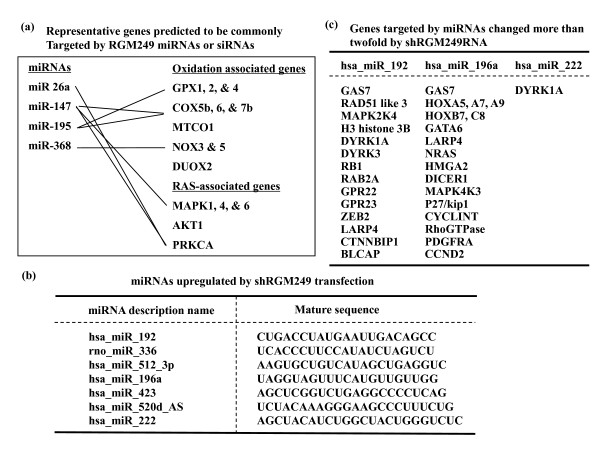
**A representative miRNA expression in microRNAarray analysis and predictive target genes**. (a) Representative genes predicted to be commonly targeted by RGM249 miRNAs or siRNAs are shown. 4 miRNAs were predicted from the RGM249 sequence by miRBase  or miRNAMAP  and it was predicted that the miRNAs might inhibit the translation of genes involved in oxidation and RAS activation. As reported previously in association with hTERT expression, PRKCA, MAPK1, and AKT1 were included in the targeted genes. (b) Seven miRNAs upregulated more than twofold in RGM249 shRNA-transfectants by microRNAarray analysis performed between transfectants with LacZ shRNA and RGM249 shRNA are shown. Some of these 7 miRNAs have been indicated in the oncogenesis of oral squamous cell carcinoma (unpublished data). (c) Representative genes targeted by miRNAs which changed more than twofold when induced by shRGM249RNA are shown. The genes were predicted mainly using miRNAMAP. Three miRNAs were presumably targeted towards miRNA processing proteins, transcriptional factors, telomere-related genes, TERT-related genes, tumor suppressor-related genes, and cell cycle-related genes.

This novel noncoding gene is strongly expressed in poorly differentiated tumor cells. The genes (mRNAs) targeted commonly by both miRNAs and siRNAs in our study were MAPK6 and PRKCA, which can induce hTERT upregulation (fig 7-(a)). Representative genes (mRNAs) targeted by miRNAs with expression upregulated more than twofold by shRGM249RNA included DICER1, high mobility group A2 (HMGA2) and GAS7, as well as hsa-miR-let 7b, 7d, 7e, 7f, and 7g targets. In particular, RGM249 might be implicated in oncogenic transformation through the inhibition of HMGA2 interacting with let-7 miRNA (fig 7-(b), (c)) [43]. RGM249 was not detected in any 'mirtrons or polycistron' detection program, genome, or miRNA database (MiRscan, PicTar, miRanda, etc.)[44]. We are now considering MED18 as a key molecule obtained from microarray analysis (fig 6 and 7) [45]. The mediator head module stimulates basal RNA pol II transcription and enables transcriptional regulation. The head subunits MED8, MED18, and MED20 form a subcomplex (MED8/18/20) with 2 submodules. The highly conserved N-terminal domain of Med8 forms one submodule that binds the TATA box-binding protein (TBP) *in vitro *and is essential *in vivo*. Beyer et al. reported that the downregulation of another mediator complex (MED28) expression in NIH3T3 cells results in a significant induction of several genes associated with smooth muscle cell (SMC) differentiation and functions as a repressor of SMC differentiation [46]. A mediator complex is essential for transcription induced by pol II in eukaryotes and is required for the recruitment of the transcription machinery after chromatin remodeling [47]. MED18 may also be involved in the repression of differentiation, transcription, and telomere elongation. Microarray analysis revealed a strong upregulation of MED18 (×24.8), the RNA polymerase II mediator which functions as a coactivator involved in transcription regulation in nearly all RNA polymerase II-dependent genes, and other RNA-related or DNA-binding genes were upregulated (fig 6). In transfectants with MED18 siRNA, both RGM249 and hTERT mRNA were significantly upregulated (Supplement fig 2-(d)). MED18 siRNA transfected into A172 cells significantly upregulated hTERT mRNA and RGM249 expression (Additional file 1-(d)). Co-transfection by both MED18 siRNA and hTERT siRNA upregulated only RGM249 mRNA, suggesting that RGM249 may functionally locate upstream hTERT mRNA, and MED18 may closely interact with RGM249. Overexpressing RGM249 is carried out for the purpose of comprehending the possible suppressive effects of miRNAs and depleting RGM249 is performed for the purpose of understanding the physiological function of this gene. Our results suggest that RGM249 may not be a precursor gene such as Onco-miR but is a Suppressor-miR and might function to lower differentiation in its expressing cells by suppressing cell growth as an independent response to hTERT expression.

## Conclusion

Taken together, the enhanced cell death in RGM249 transfectants likely resulted from an hTERT-independent mechanism or the presence of a mediator with crucial responsibility for oncogenesis, arguing that some other targets instead of hTERT might be the real players mediating these contradictory oncogenic and growth suppressive functions. It should be noted that mRNA studies do not always reflect processes at the protein level of gene regulation.

Further studies will be required to investigate these expression levels. Several ncRNAs in the exons we trapped might provide new insights into the coordinated and dynamic mechanisms of development, stress, transformation, and (de)differentiation, resulting in the possible prevention of oncogenesis and suppression of tumor progression.

## Methods

### DNA preparation and analysis

BAC clones (H-11, M-6, and J-21) were selected on the basis of results from a previous experiment (Research Genetics, Toronto, Canada) [18]. BAC DNA was isolated using a modified alkaline lysis protocol [18]. Pulsed-field electrophoresis analysis of the isolated BAC DNA was performed using the CHEF DRII system (Bio-Rad, CA, USA).

### Sequence determination and analysis, and construction of a contig of BAC clones (M-6, H-11, and J-21)

Purified BAC DNAs were shared using the AERO-MIST nebulizer (CIS-US, Bedford, MA, USA) and after ligation of adapters, these DNAs were subcloned into the BstXI sites of pSHOT II, which was constructed by subcloning the BstXI sites from pcDNA II, prior to the disclosure of the endosequences of the 3 BAC clones (M-6, H-11, and J-21) (fig 1-(b)). A sequencing library was generated by sonicating BAC and cloning it into a phosphatased EcoRV site in Bluescript; initially, each clone was sequenced using Phil Greene's Phred and Phrap programs.

### Exon trapping

For all exon trapping experiments, we used the exon-trapping system kit (Gibco-BRL, Gaithersburg MD, USA) and the supplements recommended therein. We shotgunned M-6, H-11, and J-21 into pSPL3, employing BamHI and BglII or BamHI, BglII, and PstI for 2 ways of cutting BAC DNA using restriction enzymes. This method was performed as described previously [21]. Thereafter, the BAC whole sequence or cDNA sequence was identified using a database . In 1999 to 2005, the sequenced reference human genome draft was not definitely determined on 10p14–15 and altered several times during this interval.

### Northern blotting, reverse-transcriptase polymerase chain reaction (RT-PCR), and onestep real-time RT-PCR for confirming the expression of exons in human organs

The probe, consisting of the respective exons, including two exons forming RGM249, radiolabeled by random priming using the Radprime kit (Gibco-BRL), was incubated with human multiple tissue northern blot (Human 12-Lane MTN Blot) (Clontech, Mountain View, CA, USA). One-step real-time RT-PCR experiments were performed using Human Total RNA Master Panels (Clontech) according to methods we previously reported using LightCycler (Roche, Basel, Switzerland) [48]. The primer sequences used are shown in Table 1. Each quantification was confirmed by sequencing with high reproducibility (for RGM249; fig 2-(a), r = 0.998, for hTERT) [48].

**Table 1:** Representative primer sequences used in this study

Gene	Oligonucleotide	Sequence (5'-3')
RGM249 (for RT-PCR)	sense primer	TGAGAGAATGGGTGTCCAAG
	antisense primer	CCTGGAAAGTCTAGGGATGT
RGM249 (for real-time RT-PCR)	sense primer	AGAGAATGGGTGTCCAAGAGG
	antisense primer	GGACATGTTCAGCTTCCCCTTT
hTERT	sense primer	CGGAAGAGTGTCTGGAGCAA
	antisense primer	GGATGAAGCGGAGTCTGGA
RPL22 (for RT-PCR)	sense primer	ATTGCACCCACCCTGTAGAA
	antisense primer	TGTTAGCAACTACGCGCAA
RPL22 (for real-time RT-PCR)	sense primer	GACCATCGAAAGGAGCAAGA
	antisense primer	TGTTAGCAACTACGCGCAAC
β-actin	sense primer	TCACCCACACTGTGCCCATCTACGA
	antisense primer	CAGCGGAACCGCTCATTGCCAATGG

### cDNA cloning and exon/intron organization

Using the exons identified by Northern blotting or RT-PCR, we amplified cDNA fragments from human skeletal muscle and liver "Marathon-ready" cDNA, according to the manufacturer's protocol (Clontech). Amplicons were subcloned into the pcDNA3 vector (Invitrogen, Leek, The Netherlands). A comparison of cDNA and genomic sequences revealed a total of 20 exons and detected 3 alternative splice donor sites for 6 exons.

### PCR-single-strand conformation polymorphism (SSCP) analysis for the detection of mutations in exons

To screen for point mutations in trapped exons, we performed PCR-SSCP assays using flanking intron primers, as previously described [49].

### Measurement of telomerase activity

To confirm telomerase activity in transfectants after siRNA transfection, a telomerase detection kit, TRAP-eze, (Oncor Inc., Gaithersburg, MD, USA) used for RGM249 cloning and the TeloChaser kit (TOYOBO, Osaka, Japan) used for the expression analysis of shRNA transfectants, were employed for the telomeric repeat amplification protocol assay according to the manufacturer's protocol. Quantification of TRAP was estimated using an image analyzer (CS Analyzer 3) provided by ATTO Corp. (Tokyo, Japan).

### Structural prediction of RGM249

To examine whether RGM249 can generate a protein, we performed an *in vitro *translation assay according to the manufacturer's instructions (Clontech). After examining the motif and homology ; ; ), we predicted the secondary structure of RGM249 using the Vienna RNA Secondary prediction and comparison program ; ). Subsequently, we screened the micro RNA) (miRNA) database among species. To confirm whether the RGM249 gene functions as a miRNA precursor gene, we digested the full-length RNA with a Dicer enzyme using a Dicer siRNA generation kit according to the manufacturer's protocol (Gene Therapy Systems, Inc., CA, USA).

### RGM249 mRNA expression in human organs, hepatocytes, hepatoma cell lines, and liver tissues

To confirm the transcriptional level (copy number) in human organs, liver-related normal cells, and immortalized cells, we performed one-step real-time RT-PCR using human hepatocytes (KAC, Kyoto, Japan), hepatoma cell lines (Tohoku University, Sendai, Japan), and 74 surgically resected liver tissue samples, including 33 pairs of hepatomas and corresponding adjacent tissues. We measured each copy number 3 times and calculated the average.

### Effect of RGM249 overexpression on expression of other genes in RGM249 transfectants

After ligasing a synthesized RGM249 (Operon Biotechnologies, Tokyo, Japan) into the pRNAT/U6.1/Neo/GFP vector (GeneScript Corp., Piscataway, NJ, USA) digested at the KpnI/SacI site, it was transfected into several cell lines (A172, T98G, HLF, HT1080, HeLa, SW480, Huh7, HepG2, HMc-Li7, KMST-6, SUSM-1, and NIH3T3), with NH-12 as senescence-programmed cell lines, and keratinocytes and TIG 1–20 as primary cells. Immortalized cells were transfected with FuGene (Roche, Basel, Switzerland) to obtain stable cells overexpressing RGM249. After enhanced green fluorescent protein expression in the presence of a neomycin-resistant gene in all cell types was confirmed, β-gal staining (Senescence Detection kit; BioVision, CA, USA) and RT-PCR were performed. Primary cultured cells were electroporated using Nucleofector technology (Amaxa AG, Cologne, Germany) under optimal conditions. At least 5 transfectants were examined in mock or RGM249 transfectants. The respective gene expression (GE) ratio to β-actin mRNA in mocks or RGM249 tranfectants was analyzed using the Mann-Whitney test. Between both transfectants, FACS analysis of HLF cells was performed using Guava EasyCyte Mini according to the manufacturer's instructions (GE Healthcare UK Ltd., Buckinghamshire, England).

### Transfection of total dsRNA and gene-specific siRNA derived from the RGM249 gene

dsRNA derived from the RGM249 sequence was generated from the expression vector with RGM249 in the MCS of Litmas28i (NEB, MA, USA) using an *in vitro *transcription method (T7 RiboMAX Express Large Scale RNA Production System, Promega, Madison, WI, USA) and was transfected into 4 hepatoma cell lines (HLF, Alexander, HepG2, and HMc-Li7) at a concentration range of 10–40 μg/ml medium. Subsequently, to avoid unspecific inhibition of dsRNA through interferon (INF) activation [50,24], stealth siRNAs were designed on-line using RNAi designer provided by Invitrogen (Tokyo, Japan). At the optimal concentration (25 nM) of siRNA to the recipient (HLF), which expresses it strongly, we repeatedly performed transfections to examine the correlation of telomerase-related factors (hTR, TEP1, dyskerin, RPL22, etc.) with RGM249 expression [31]. The expression of telomerase-related genes was estimated by RT-PCR after transfection of dsRNA (fig 4-(a)).

### siRNA method

Two kinds of siRNAs (siRNA ① and siRNA ②) were designed using the Stealth RNAi designer and synthesized by Invitrogen (Invitrogen Corp, Carlsbad, CA, USA). The sequences are shown in Table 2. To suppress RGM249 expression with siRNA, we performed the following transfection procedures in HLF cells: transfection using Stealth siRNA ①, transfection employing Stealth siRNA ②, and transfection with both Stealth siRNA ① and Stealth siRNA ② at a final concentration of 25 nM as an optimal condition. We collected transfectants 24 hours after transfection for RNA extraction and confirmed the inhibitory effect on RGM249 expression by one-step RT-PCR. HLF cells treated with FuGene Reagent without siRNAs and with Stealth LacZ siRNA were used as controls. Transfectants were collected 48 hours, 1 week, and 2 weeks after transfection. Subsequently, we confirmed the suppressive effect of shRNA with the same functional sequence as that of siRNA on RGM249 expression, using the BLOCK-iT RNAi

**Table 2:** siRNA and shRNA sequence used in this study

Stealth siRNA ①	Sequence (5'-3')
top strand	UUCCCUUUGUCUAGCCUUAUUCUGC
bottom strand	GCAGAAUAAGGCUAGACAAAGGGAA
Stealth siRNA ②	
top strand	AUACUGUGAGAUUCGAGGAUCAUAC
bottom strand	GUAUGAUCCUCGAAUCUCACAGUAU
RGM249 shRNA	Sequence (5'-3')
top strand	CACCGCAGAATAAGGCTAGACAAAGCGAACTTTGTCTAGCCTTATTCTGC
bottom strand	AAAAGCAGAATAAGGCTAGACAAAGTTCGCTTTGTCTAGCCTTATTCTGC
RGM249 mt-1 shRNA	
top strand	CACCGCAGAATAAGGCTAGACAAAGCGAACTTTGTCAGCCTTATTCTGC
bottom strand	AAAAGCAGAATAAGGCTGACAAAGTTCGCTTTGTCTAGCCTTATTCTGC
RGM249 mt-2 shRNA	
top strand	CACCGCAGAATAAGGCTAGACAAAGCGAACTTTGTCTAGCCTTATTTGC
bottom strand	AAAAGCAAATAAGGCTAGACAAAGTTCGCTTTGTCTAGCCTTATTCTGC

Expressing vector (Invitrogen) which was designed to generate shRNA in transfectants. To examine the effect on hTERT expression 7 to 10 days later after selection with 200 μg/ml Zeocin, an shRNA-generating lentiviral vector with the functional sequence of RGM249 (fig 3-(b)) was transfected. Since the mock of the lentiviral vector was linearized according to the manufacturer's instructions, we used transfection reagents without RNA and the vector, LacZ shRNA as the controls, and wtRGM249 or mtRGM249 (mt-1 or mt-2) with one base (T or C) deletion in the wtRGM249 sequence. We evaluated GE in the transfectants semiquantitatively. Stealth siRNA and shRNA sequences for RGM249 are shown in Table 2.

### Gene expression profile by microarray analysis

Microarray analysis was performed for total RNA purified from transfectants with LacZ shRNA and from those with RGM249 shRNA following confirmation of the downregulation of both RGM249 and hTERT mRNA expression by RT-PCR after strict quality control in accordance with the guidelines of the Hokkaido System Science (Sapporo, Japan). Data for genes that had changed more than twofold were additionally analyzed using Gene Ontology Tree Machine  and typical GE changes are shown in fig 6 and the left fig in Additional file 2. miRNA array analysis was performed using the same samples as the microarray analysis samples (Filgen, Inc., Nagoya, Japan). The results are shown in fig 7(b) and the right fig in Additional file 2. Genes targeted by miRNAs were investigated using DIANA-microT, Pictar, TargetScan, and miRanda .

## Abbreviations

hTR: telomerase RNA component; BAC: Bacterial artificial chromosome; hTERT: human telomerase reverse transcriptase; siRNA: short interfering RNA; shRNA: short hairpin RNA; RPL22: ribosomal protein L22; ncRNA: noncoding RNA; GE: gene expression.

## Competing interests

The authors declare that they have no competing interests.

## Authors' contributions

MN and SR carried out the molecular genetic studies. WJ and DH participated in the sequence alignment and drafted the manuscript. MJ kindly provided the exon trapping vector. SG kindly provided the clinical specimens. MN and VA participated in the study design and performed the statistical analysis. Other authors conceived of the study, and participated in its design and coordination and helped to draft the manuscript. All authors read and approved the final manuscript. All contributors who do not meet the criteria for authorship are listed in Acknowledgements.

## Supplementary Material

Additional file 1 **A comparison of hTERT mRNA expression between mock and RGM249 transfectants.** In response to upregulated RGM249 mRNA, hTERT mRNA did not induce any significant change in senescence-programmed cells (keratinocytes, TIG1-20, and NH-12) except for KMST-6 cells. The dotted line indicates hTERT mRNA expression in parental cells. Six transfectants were examined in both mock and RGM249 transfectants. (b) Actin mRNA and RPL22 mRNA showed no significant difference among untransfected cells and mock or RGM249 transfectants in the 4 cell lines described in (a). (c) Comparison of hTERT mRNA expression between mock and RGM249 transfectants. In response to upregulated RGM249, hTERT mRNA induced a significant change in suppression in (left) HLF cells (poorly differentiated hepatoma) and (right) A172 cells (glioblastoma). The dotted line indicates mean mRNA expression in untransfected parental cells. hTR mRNA and b-actin mRNA showed no significant differences in b-actin, hTERT, RGM249, and hTR among untransfected cells and mock or RGM249 transfectants. (d) By transfection of MED18 siRNA or MED18/hTERT siRNA into A172 cells, an insight into the regulative network among 3 genes (RGM249, MED18, and hTERTmRNA) could be obtained. The number of respective inductions was more than 5. Following transfection of MED18 siRNA, both TERT mRNA and RGM249 mRNA were significantly upregulated (P < 0.01). Following co-transfection of MED18 siRNA and TERT siRNA, RGM249 mRNA was significantly upregulated (P < 0.05). Click here for file

Additional file 2 **A representative case of microarray analysis and microRNAarray analysis.** Microarray analysis (a) and miRNA array analysis (b) were performed for total RNA purified from transfectants with LacZ shRNA and from those with RGM249 shRNA. The representative analyses are demonstrated, respectively. Click here for file
