# Supplementary figures and images for: A noncoding RNA gene on chromosome 10p15.3 may function upstream of hTERT
Source: BMC Mol Biol. 2009 Feb 2;10:5. doi: 10.1186/1471-2199-10-5 (PMC2661890; doi:10.1186/1471-2199-10-5)

# Additional file 1

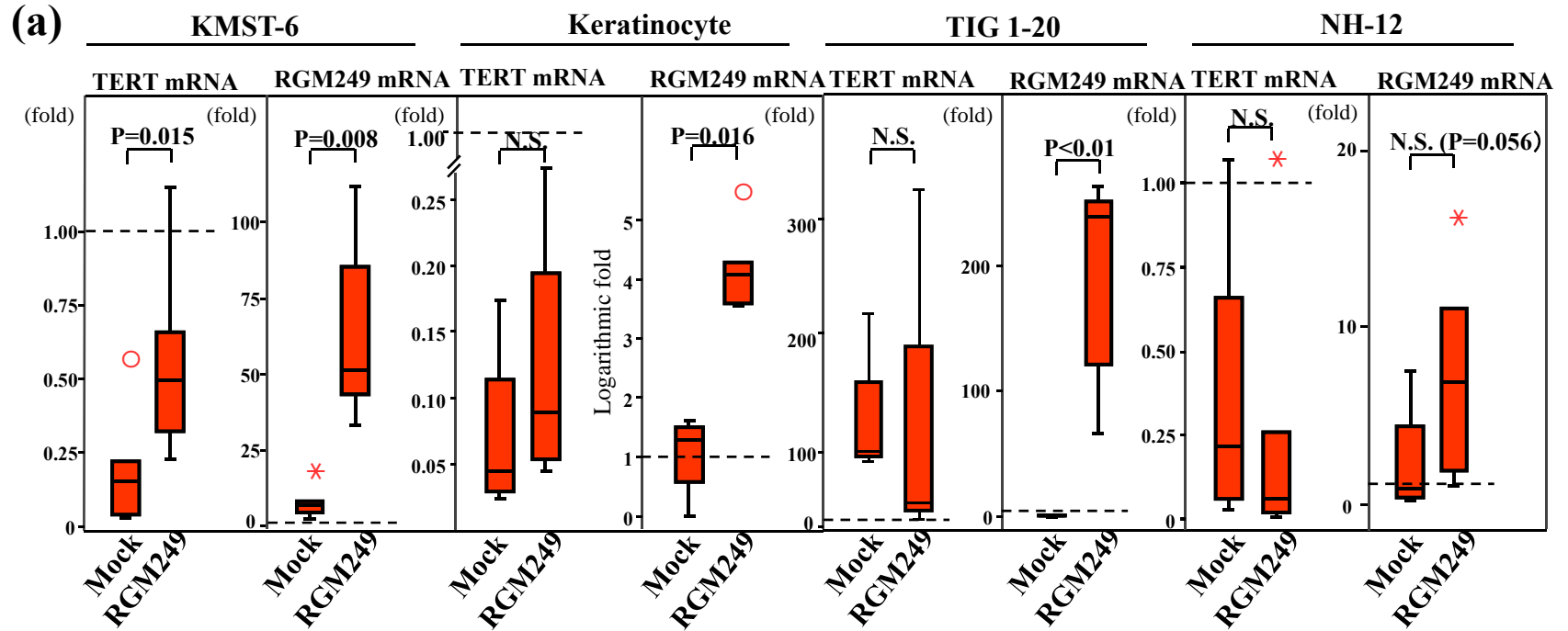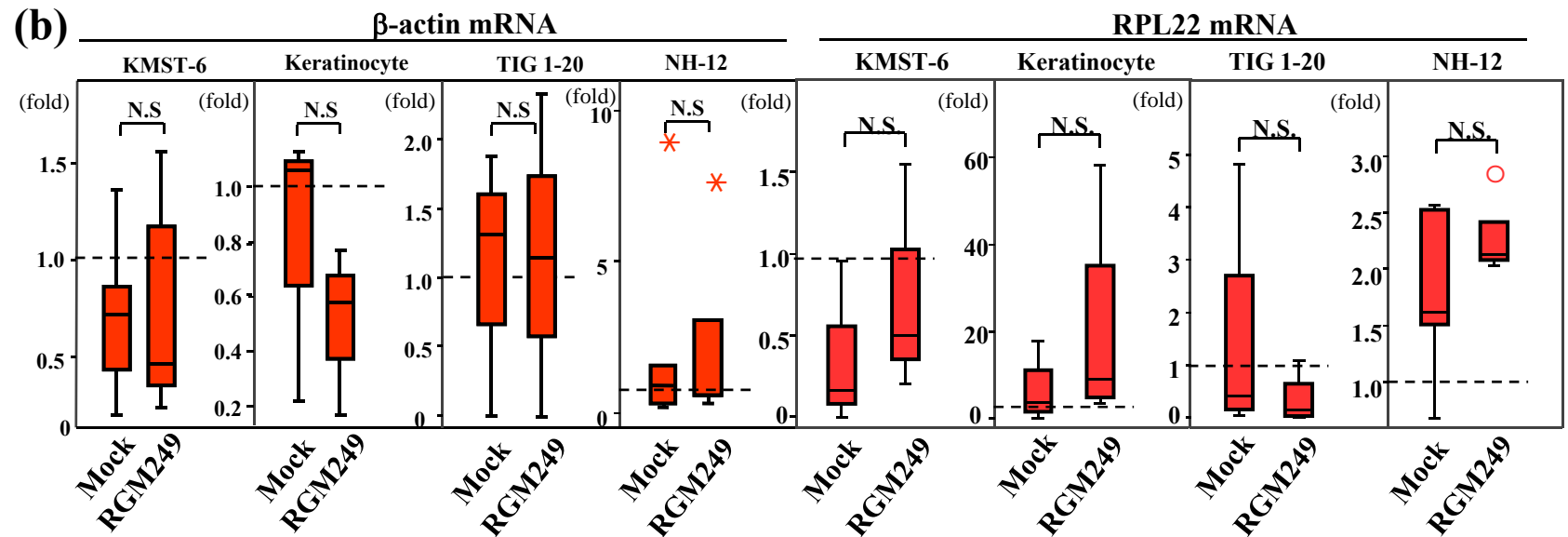

**(c)****GE in HLF cells (poor differentiation)****GE in A172 cells (poor differentiation)**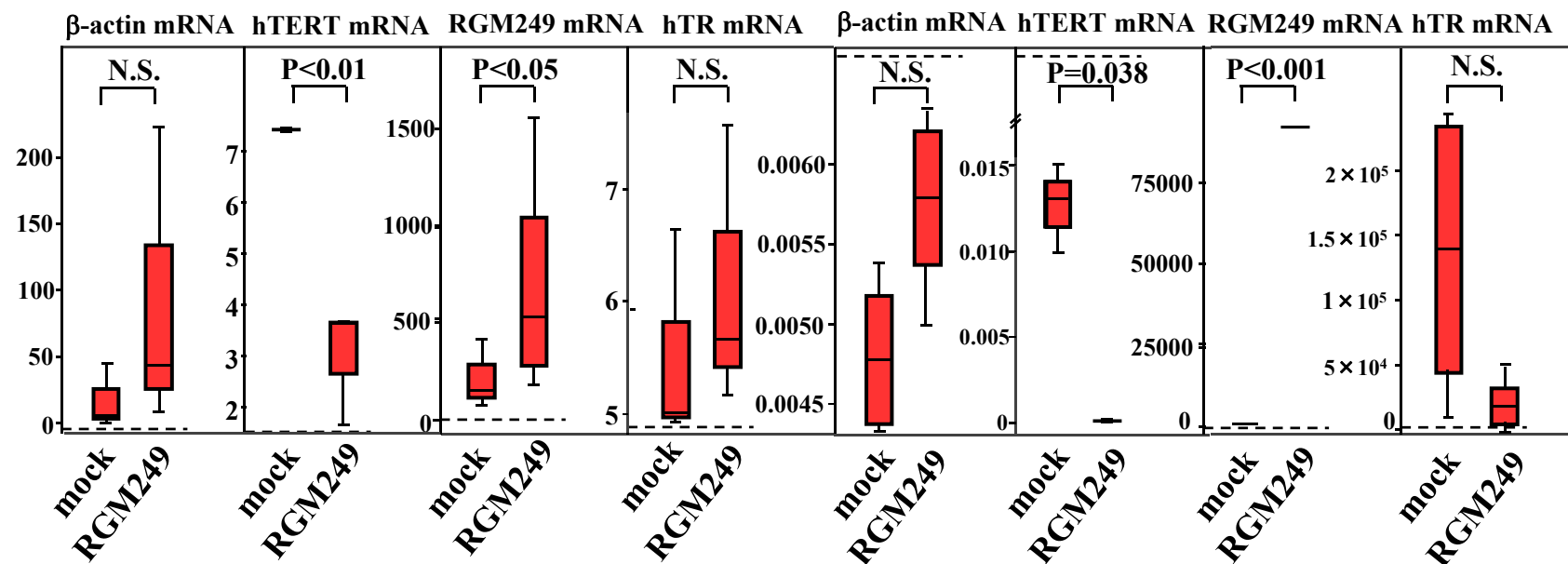**(d)**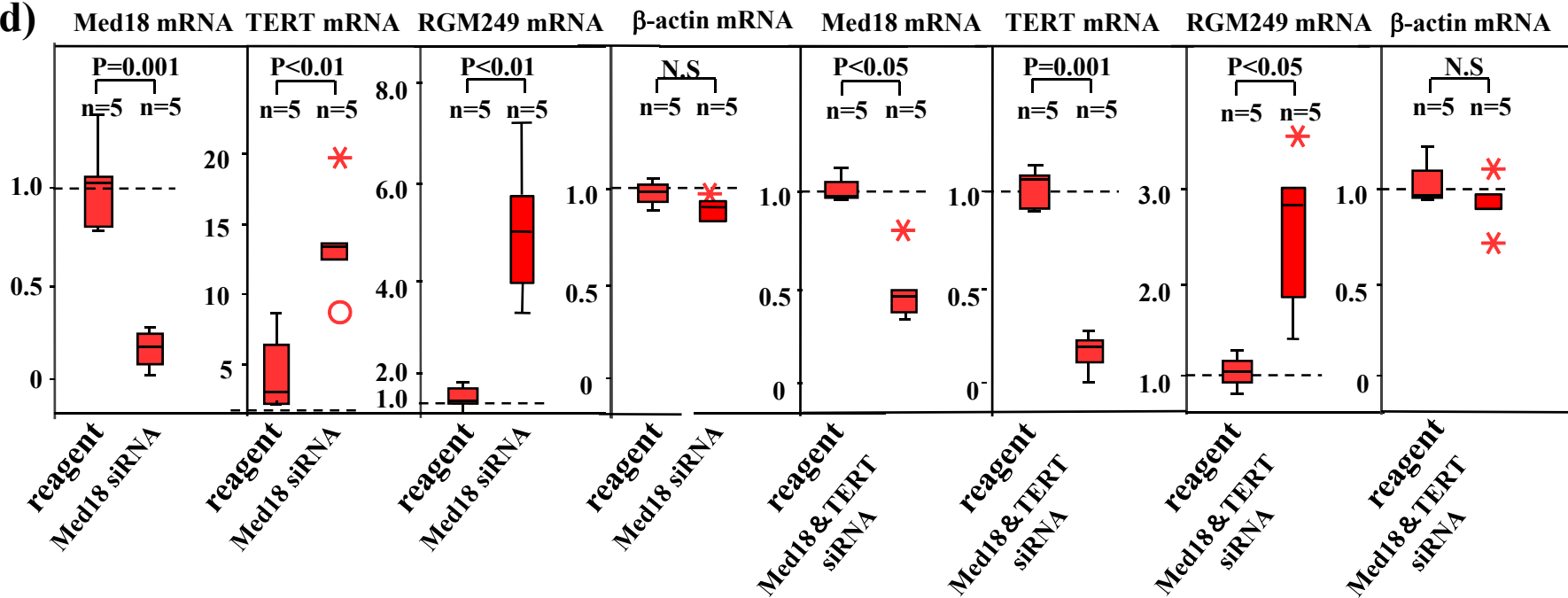

Supplement: Additional file 1 — A comparison of hTERT mRNA expression between mock and RGM249 transfectants. In response to upregulated RGM249 mRNA, hTERT mRNA did not induce any significant change in senescence-programmed cells (keratinocytes, TIG1-20, and NH-12) except for KMST-6 cells. The dotted line indicates hTERT mRNA expression in parental cells. Six transfectants were examined in both mock and RGM249 transfectants. (b) Actin mRNA and RPL22 mRNA showed no significant difference among untransfected cells and mock or RGM249 transfectants in the 4 cell lines described in (a). (c) Comparison of hTERT mRNA expression between mock and RGM249 transfectants. In response to upregulated RGM249, hTERT mRNA induced a significant change in suppression in (left) HLF cells (poorly differentiated hepatoma) and (right) A172 cells (glioblastoma). The dotted line indicates mean mRNA expression in untransfected parental cells. hTR mRNA and b-actin mRNA showed no significant differences in b-actin, hTERT, RGM249, and hTR among untransfected cells and mock or RGM249 transfectants. (d) By transfection of MED18 siRNA or MED18/hTERT siRNA into A172 cells, an insight into the regulative network among 3 genes (RGM249, MED18, and hTERTmRNA) could be obtained. The number of respective inductions was more than 5. Following transfection of MED18 siRNA, both TERT mRNA and RGM249 mRNA were significantly upregulated (P < 0.01). Following co-transfection of MED18 siRNA and TERT siRNA, RGM249 mRNA was significantly upregulated (P < 0.05). [file 1471-2199-10-5-S1.pdf]

## Additional file 2

(a) **Microarray analysis**

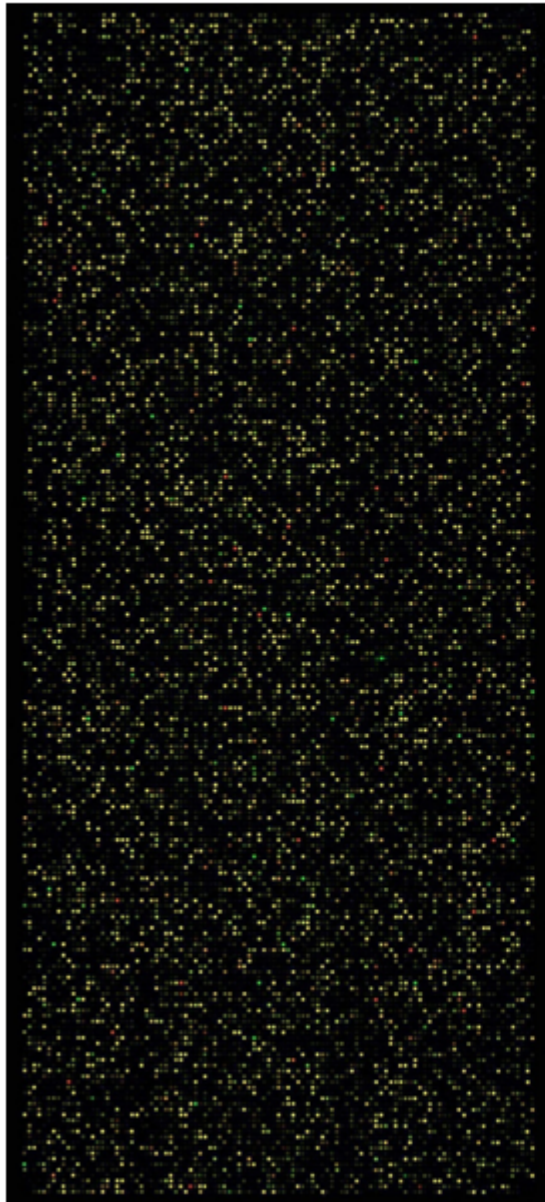

(b) **MicroRNAarray analysis**

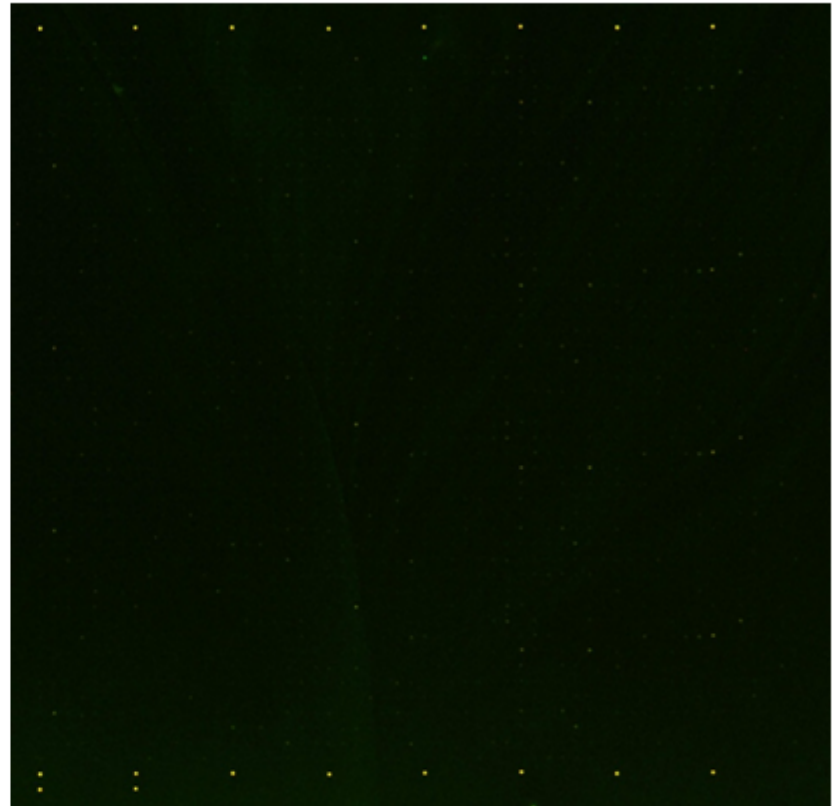

Supplement: Additional file 2 — A representative case of microarray analysis and microRNAarray analysis. Microarray analysis (a) and miRNA array analysis (b) were performed for total RNA purified from transfectants with LacZ shRNA and from those with RGM249 shRNA. The representative analyses are demonstrated, respectively. [file 1471-2199-10-5-S2.pdf]
